# Assessments of Birth Outcome of Twin Delivery and Associated Factors among Newborns in Dessie Referral Hospital, Dessie, Ethiopia, 2019

**DOI:** 10.1155/2021/2421843

**Published:** 2021-03-16

**Authors:** Nigusie Abebaw, Mohammed Abdu, Natnael Girma

**Affiliations:** ^1^Samara University, Samara, Ethiopia; ^2^Wollo Univesity, Dessie, Ethiopia; ^3^Wollo University, College of Health Science, Dessie, Ethiopia

## Abstract

**Background:**

There was a fast improvement of twin's birth outcomes in the past decade, but it was average in developing countries. Stillbirth, preterm birth, low birth weight, and birth asphyxia are the major contributors to poor twin birth outcomes. This study was crucial to address the gaps and clarify the outcome of twin delivery.

**Objectives:**

To assess the birth outcome of twin delivery and associated factors among newborns who were delivered in Dessie Referral Hospital, Ethiopia, 2019.

**Methods:**

Institutional-based retrospective cross-sectional study was employed among 385 maternal records from Nov 10/2013 to Dec 10/2019. Data were selected by using a random sampling technique. Frequencies, proportion, and summary statics were used to describe the study population. The data were entered into Epi Info and exported in the SPSS version 20 for analysis. All variables with *p* value < 0.20 in bivariable logistic regression analysis were considered for multivariable logistic regression analysis; adjusted odds ratio with 95% confidence interval was used to measure the association variable with *p* value < 0.05 which was statistically significant.

**Results:**

This finding showed that the prevalence of twin birth outcome accounts 23.4% (95 % CI, 19.2–27.5). Low birth weight 9.1%, stillbirth 4.2%, Apgar score < 7 9.1%, and neonatal death 1 % were accounted. Hypertension disorder (95% CI, 6.01(2.43–14.87)), rural residence (95% CI 2.46(1.39–4.37)), PROM (95% CI 6.39(2.52–16.16)), and no ANC follow-up (95% CI, 13.47(2.49–72.85)) were significantly associated with adverse twin birth. *Conclusions and Recommendations.* Magnitude of twins' adverse birth outcome was 23.4%. Hypertension disorder, rural residence, PROM, and no ANC follow-up were significant variables for twins' adverse birth outcome. Therefore, all healthcare providers should give sustainable educations and instructions about the importance of sticking with the recommended ANC follow-up.

## 1. Introduction

Twin birth is a type of multiple births when the mother gives birth to two offspring from the same pregnancy. It can be either dizygotic or monozygotic. Twin pregnancies are risks during pregnancy, labour, and delivery as well as during postnatal period as the result of preterm delivery, antepartum haemorrhrage, chronic disease, and postpartum haemorrhage [1]. Adverse birth outcomes are the most important vital statistics used to assess maternal and child health program. They are indicators of the quality of antenatal care, medical services, and general health services to the mother and the children [2, 3].

In Ethiopia, National Reproductive Health Strategy adopted in Ethiopia showed that increased number of midwives and surgeon prepare health center with basic obstetric and new born equipments, equip all hospitals to deliver comprehensive obstetric and new born care, improve antinatal care and promote institutional delivery, ensure availability of medicine, postpartum and new born care, improve referral and health care financing systems to expand positive birth outcome during labour delivery [1, 4].

Twin pregnancy is among the obstetric conditions with accounts of 2 to 4% of total births, with a prevalence ranging from 0.9 to 2.4% in Brazil. It is one of the increasing risks of perinatal mortality, a well predictable factor. Twin pregnancy is negative impact from a complex interaction of genetic and environmental determinants occurring in approximately 2–4% of live births and remarkably rates are highest in some parts of Africa, especially in Ethiopia, where health care is not well established. However, its prevalence increased more than 70% globally in the last three decades mainly in high- and middle-income countries [1, 5, 6].

Adverse birth outcomes among twin deliveries, such as prematurity, low birth weight, and birth defects, represent significant problems in both developing countries. Each year, about 15 million babies in the world, more than one in 10 births, are born too prematurely [7–9]. More than one million of those babies die shortly after birth; countless others suffer from lifelong physical, neurological, or educational disabilities, often at great cost to families [10–12].

Adverse birth outcome for twin delivery is a critical health issue in developing countries such as Ethiopia. It resulted in many bad consequences, neonatal and infant morbidity and mortality. More than a million of them die immediately after birth; many others suffer from lifelong physical, neurological, or educational disabilities. According to Ethiopian Demographic and Health Survey in 2016, there were high rates of twin prenatal mortality during parturition period. From the total deliveries, thirty-three deaths per 1000 live births were reported [13–15].

There are factors to increase the negative birth outcome of twin delivery; from these, some of them are associated with increased maternal age, primiparity, low birth weight, chronic disease, low ANC follow-up, and PROM [16, 17].

Twin pregnancy has increased the rate of obstetric and perinatal complications such as preterm labour, preachalasia, and postpartum haemorrhage, and therefore, the aim of this study is to assess the adverse birth outcome of twin deliveries and its determinant factor, the birth outcome in Dessie Referral Hospital during the study period.

## 2. Method and Materials

### 2.1. Study Area and Period

The study was conducted from Nov 10–Dec 10, 2019, in Dessie Referral Hospital which is located in Amhara region, Ethiopia, 401 km far from the capital city of Addis Ababa. Based on 2007 Ethiopian Census conducted by Central Statistical Agency, Dessie has the total population of 250,536. Of them, 117,060 are men and 78,242 are women [18]. Dessie Referral Hospital is one of the largest hospitals in Amhara region, which provides all-patient diagnosis and treatment services for the communities. The hospital serves more than 4 million people including other neighborhood regions such as Afar and Oromia regional state. The hospital has 456 healthcare providers; among them, 267 were males and 188 females. They are a total of 24561 deliveries per year; among them, 748 were twin deliveries.

### 2.2. Study Design

Institutional-based retrospective cross-sectional study design was employed among 385 maternal records from Nov 10/2013 to Dec 10/2019.

### 2.3. Source of Population

The source of population of this study was total twin delivery records of Dessie Referral Hospital from November 10/2013 to December 10/2019.

### 2.4. Study Population

The study population of this research was all-twin delivery recorded in Dessie Referral Hospital from September 01/2013 to September /01/2019. Maternal medical record sheet was used to review this study in 6 years recorde sheet before the study period.

### 2.5. Eligibility Criteria

#### 2.5.1. Inclusion Criteria

The study included all twin deliveries recorded from September 01/2013–September 01/2019.

#### 2.5.2. Exclusion Criteria

Twin deliveries were recorded with incomplete recorded information sheets.

### 2.6. Sample Size Calculation

The sample was calculated by using single population formula, 95% confidence interval, and 5% margin of error and proportion rate of 50% of adverse twin birth outcome rate

(1) $$\begin{array}{c} n=\frac{\left(z{\left(a/2\right)}^{2}\cdot p\left(1-p\right)\right)}{d^{2}}, \end{array}$$

where *n* = minimum sample size, z (a/2) = desired level of confidence interval 95% (1.96) *P*=50% proportion rate, and *d* = margin of error 5% (0.05). The total sample size becomes *n* = 384.

### 2.7. Sampling Procedure

The eligible record was selected by the investigator and the health management information system (HMIS) focal person of Dessie Referral Hospital before data collection. The records were selected by using simple random sampling technique (lottery method) to gather the exact sample size. From September 2013–September 2019, the total deliveries of Dessie Referral Hospital were 24561. Among them, 748 were twins; therefore, by using the simple random sampling method, 384 maternal records were selected to conduct this research.

### 2.8. Data Collection Procedure

Pretest was performed at Borumeda Hospital and structured checklist was developed after reviewing relevant literatures. The checklist was designed to obtain relevant information on the predictor variables such as demographic, obstetric, maternal complication, intervention, and component of modified WHO partograph The conditions of the baby such as Apgar score were assessed and Apgar score = 7 was considered as satisfactory by this study [19]. Five midwifes participated for data collection. All records were securitized for all variables to extract the maternal medical records and integrated maternal and new born medical card.

### 2.9. Variables

#### 2.9.1. Dependent Variables

Dependent variables were twin birth outcomes.

#### 2.9.2. Independent Variables

*(1) Demographic Factors*. Age, gravidity, parity, and residence.

*(2) Obstetric Factors*. NC history, mode of delivery, gestational age at onset if labour, outcome of delivery, and sex of the new born.

*(3) Partograph-Related Factors*. Quality of the partograph (standard, substandard), time of action line closed, and time of action taken based on the partograph result.

### 2.10. Data Quality Control

For data collectors and supervisors, 1 and a halve day training was given about data collection system. The data collectors were supervised by a supervisor daily and reported to the principal investigator every day; before the actual data collection, the checklist was pretested on 5% of the sample size, which are 20 maternal medical records in Borumeda Hospital, after that unclear questioners were revised, and the actual questioner was prepared in English and translated into Amharic and then translated back into English to check the exact adjustment of the questioner.

### 2.11. Data Processing and Analysis

All checklists were checked for their completeness and consistency, and double data entry was made using Epi Info 7 software. Then, the data were exported into SPSS version 20 for analysis.

Frequencies, proportion, tables, and summary statistics were used to describe the study population in relation to the relevant variable.

Bivariable analysis was carried out and the variable with P value less than 0.20 was titled to multivariable logistic regression significantly associated with twin birth outcome. Finally, adjusted odds ratio with its 95% confidence interval was used to measure the association variable with P value less than 0.05 statistical associations.

### 2.12. Ethical Consideration

Ethical clearance was obtained from the review committee of Wollo University, College of Health and Medical Science. The permission and agreement consent were obtained from Dessie Referral Hospital before starting of the study. Mandatory information was collected by the trained data collector with keeping the confidentiality of the maternal medical history.

## 3. Results

### 3.1. Socio-Demographic Characteristics

In this study, a total of 385 study participants/maternal record were involved. From the total number of maternal records, mean age was 29.78 with Std. deviation of ±6.53. Regarding the sex of fetus, 195 (50.6%) were female. Majority of respondents, 371 (96.4%) of the study participants, were married. From the total records, 131 (34.0%) of participants, were Muslim religion followers and regarding residence, more than half (58.2%) of the participant residence was rural (Table 1).

### 3.2. Obstetric Complications, Fetal Conditions, and Other Intrapartum-Related Variable

This finding showed that only 34 (8.8%) respondents have intact membrane status. Majority of mothers have regular ANC follow-up, 369 (95.8%). Regarding gestational age at onset of labour, 327 (84.9%) of mothers were found between weeks of 37–42. Majority of the mothers about previous index pregnancy were good 306(79.5%). Regarding bleeding disorder during pregnancy and after delivery, APH accounts for 18 (4.7%) and 11 (2.9%) were affected by PPH, respectively (Table 2).

#### 3.2.1. Partograph-Related Characteristics

About two-third of respondents, 263 (68.3%), have got action according to line. Based on WHO partograph standard ,311(80.8) did not fulfill the partograph recording criteria. Almost three-fifth, 313 (81.3%), of the respondents' duration of labour were normal based on the partograph standard.

#### 3.2.2. Magnitude of Twin Birth Outcome

**Figure 1:**
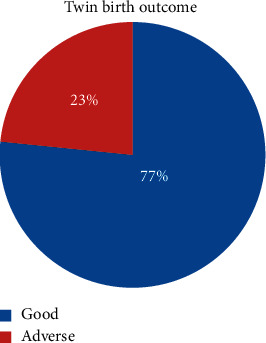
Magnitude of adverse birth outcome in Dessie Referral Hospital, Northern Ethiopia, 2019 (*n* = 385).

Out of 385 participants, 90 (23.4%) (95 % CI, 19.2%–27.5 %) of them have adverse birth outcome of twins while the remaining 295 (76.6%) had good birth outcome. From the total finding, low birth weight 35(9.1%), still birth 16 (4.2%), Apgar score < 7 35 (9.1), and neonatal death 1% were accounted (Figure 1 and Table 3).

#### 3.2.3. Factors Associated with Adverse Birth Outcome

Bivariable analysis result showed that hypertension disorder, residence, age, ANC, membrane status, APH, and PPH were significantly associated with adverse birth outcome.

In multivariable analysis, hypertension disorder, residence, PROM, and regular ANC follow-up were identified to be significantly associated with adverse birth outcome.

Mothers who had hypertension disorder were (AOR = 6.01, 95% CI (2.43–14.87)) six times more likely to develop adverse birth outcomes compared to mothers who had no hypertension disorder. Mothers who come from rural areas were two times more likely to develop adverse birth outcome as compared to mothers who come from urban areas (AOR = 2.46, 95% CI (1.39–4.37)). Mothers who follow ANC regularly were thirteen times more likely to have adverse birth outcome as compared to their counterpart ((AOR = 13.4795% C (2.49–72.85)).

Mothers who had normal membrane status were six times more likely to develop adverse birth outcome as compared to mothers who had normal membrane status ((AOR = 6.39, 95% C (2.52–16.16)) (Table 4).

## 4. Discussion

The findings of this study showed that adverse birth outcome of twin's delivery was nearly one-fourth (23.4%) (95 % CI, 19.2%–27.5 %). Low birth wt. 9.1%, still birth 4.2%, Apgar score < 7 9.1%, and neonatal death 1 % were accounted. Among factors, hypertension disorder, rural residence, PROM, and no ANC follow-up were identified to be significantly associated with adverse birth outcome. The prevalence of adverse birth outcome in this study is in line with the result of the studies conducted in Hosanna (24.5%) [16], but the finding was lower than studies done in Gonder University Hospital (32.6%) [7]. The reason for the difference in this finding was due to the difference in study design,socio demographic characteristics of the participants.

In this study, mothers who had hypertension disorder were six times more likely to be associated with adverse birth outcome as compared to mothers who had no hypertension disorder. This finding was in line with other studies done in Hawasa [13] and low- and middle-income countries [8]. The reason for this might be due to the fact that complications of hypertension during pregnancy can cause impaired placentation and placental ischemia.

Mothers who come from rural areas were two times more likely to be associated with adverse birth outcome as compared to mothers who come from urban areas. This might be due to the different educational level and in-depth of knowledge of the patient, it is clearly known that living in rural areas lead to decrease in exposure to information that is unable to understand the disease process and effect of adverse outcome. The finding of this study vary with study conducted in Suhul Shire Hospital. This difference is due sociodemographic characteristics of the participants [2]. The variations between the findings may be attributable to the variations in methodological and socio-economic variations. ANC follow-up regularly is important strata to identify negative impact of the birth outcome and its determinate factors.

Mothers who do not have regular ANC follow-up were thirty times more likely to have adverse birth outcome as compared to their counterpart; this finding was in line with the study done in Gonder University [7] and Hawasa [13]. This may be due to the fact that not getting adequate counseling and health education given by healthcare providers about disease prevention and its complication during pregnancy.

This finding showed that the mothers becoming PROM neonatal outcome were six times more likely to be associated with adverse birth outcome as compared to mothers who had normal membrane status, the finding of this study was a similar study done in Suhul Hospital Shire [2]. The reason for this might be due to the fact that PROM can cause infection in both the fetus and newborns which leads to bad neonatal outcome during childbirth.

## Figures and Tables

**Table 1:** Frequency distribution of participant socio-demographic variables in Dessie Referral Hospital, Northern Ethiopia, 2019 (*n* = 385).

Variable	Category	Frequency	Percent
Sex of fetus	Male and male	131	34.0
	Male and female; female and female	190	49.4
		64	16.6

Age in year	18–28	119 30.9	30.9
	29–39	175 45.5	45.5
	40–50	91	23.6

Marital status	Single	14	3.6
	Married	371	96.4

**Table 2:** Distribution of obstetric complications, fetal conditions, and intrapartum-related variables, Ethiopia, 2019 (*n* = 385).

Variable	Category	Frequency	Percent
Membrane status	Normal	351	91.2
	PROM	34	8.8

Parity	Multi	249	64.7
	Primi	136	35.3

ANC history	Following ANC regularly	369	95.8
	Not following ANC	16	4.2

Gestational age at labour	<37	32	8.3
	37–42	327	84.9
	>42	26	6.8

Prior outcome to index baby	Good	306	79.5
	Adverse	79	20.5

Duration of labour	Normal; prolonged	313, 72	81.3, 18.7

Mode of current delivery	SVD	247	64.2
	Instrumental	56	14.5
	C/S	82	21.3

Maternal complication	Yes	117	30.4
	No	268	69.6

Maternal death	Yes	1	0.3
	No	384	99.7

APH	Yes	18	4.7
	No	367	95.3

PPH	Yes	11	2.9
	No	374	97.1

Neonatal death	Yes	4	1.0
	No	381	99.0

Hypertensive disorder	Yes	41	10.6
	No	344	89.4

**Table 3:** Patterns of adverse birth outcome in Dessie Referral Hospital, Northern Ethiopia, 2019 (*n* = 385).

Variable	Category	Frequency	Percent
Low birth weight	Yes	35	9.1
	No	350	90.9

Still birth	Yes	16	4.2
	No	369	95.8

Apgar score < 7	Yes	35	9.1
	No	350	90.9

Neonatal death	Yes	4	1.0
	No	381	99.0

Total neonatal adverse outcome	Yes	90	23.4
	No	295	76.6

**Table 4:** Factors associated with adverse birth outcome in Dessie Hospital, Northern Ethiopia, 2019 (n=385).

Variable	Category	Birth outcome		COR (95% CI)	AOR (95% CI)
		Adverse	Good		
Hypertension disorder	Yes	30	11	12.90 [6.12 − 27.18]^*∗∗∗*^	6.01, [2.43 − 14.87]^*∗∗*^
	No	60	284	1.00	1.00

Residence	Urban	37	187	1.00	1.00
	Rural	53	108	2.48 [1.53 − 4.01]^*∗∗∗*^	2.46 [1.39 − 4.37]^*∗∗*^

Age	18–28	79	40	1.00	
	29–39	140	35	1.80 [0.79 − 4.10]	
	40–50	76	15	1.24 [0.56 − 2.74]	

ANC	Following regularly	76	293	1.00	1.00
	Not following	14	2	26.98 [6.0 − 121.3]^*∗∗∗*^	13.47 [2.49 − 72.85]^*∗∗*^

PROM	No	65	286	1.00	
	Yes	25	9	12.22 [5.44 − 27.42]^*∗∗∗*^	6.39 [2.52 − 16.16]^*∗∗∗*^

PPH	Yes	7	4	6.13 [1.75 − 21.46]^*∗∗∗*^	0.52 [0.048 − 5.72]
	No	83	291	1.00	1.00

APH	Yes	15	3	19.46 [5.49 − 68.99]^*∗∗∗*^	5.19 [0.80 − 33.65]
	No	75	292	1.00	1.00

^*∗*^ *P*  < .05, ^*∗∗*^*P*  < .01, and ^*∗∗∗*^ value < 0.001 probability value; CI, = confidence interval; COR, = crude odds ratio; AOR, = adjusted odds ratio.

## Data Availability

The datasets used and analyzed during the current study are available from the corresponding author on reasonable request.
